# Unraveling the immune mechanisms and therapeutic targets in lung adenosquamous transformation

**DOI:** 10.3389/fimmu.2025.1542526

**Published:** 2025-06-03

**Authors:** Haiyan Xu, Ying Yang, PingLi Wang, Shengnan Lin, Xiaochun Zhang, Huiwen Ni, Zhiyong Xu

**Affiliations:** ^1^ Department of Biobank, The Second Affiliated Hospital of Zhejiang University School of Medicine, Hangzhou, China; ^2^ Department of Respiratory and Critical Care Medicine, The Fourth Affiliated Hospital of Zhejiang University School of Medicine, Yiwu, China; ^3^ Department of Respiratory and Critical Care Medicine, The Second Affiliated Hospital of Zhejiang University School of Medicine, Hangzhou, China; ^4^ Breast Cancer Center, Zhejiang Cancer Hospital, Hangzhou Institute of Medicine, Chinese Academy of Sciences, Hangzhou, China

**Keywords:** adenosquamous lung carcinoma (ASLC), adenocarcinoma-to-squamous cell carcinoma transformation (AST), tumor immune microenvironment (TIME), tumor-associated macrophages (TAMs), tumor-associated neutrophils (TANs)

## Abstract

Adenocarcinoma-to-squamous cell carcinoma transformation (AST) induces drug resistance in patients with lung adenocarcinoma (LUAD), often resulting in unfavorable clinical outcomes. In recent years, it has been found that alterations in the tumor immune microenvironment (TIME) during adenosquamous carcinoma trans-differentiation also influence the efficacy of immunotherapy. Moreover, the aberrant expression and activation of several driver genes for AST lead to abnormal infiltration and function of immune cell by remodeling the cellular inflammatory phenotype. In this review, we will systematically present the changes in the TIME and molecular regulatory mechanisms during adenosquamous carcinoma differentiation, aiming to gain a better understand of the function of immune cells during this process and the potential value of combining immunotherapy to enhance the treatment of non-small cell lung cancer (NSCLC).

## 1 Introduction

Non-small cell lung cancer (NSCLC) accounts for approximately 85% of all lung cancers, with lung adenocarcinoma (LUAD) and squamous cell carcinoma (LUSC) being the two most common subtypes. The phenomenon of adenocarcinoma-to-squamous cell carcinoma transformation (AST) has gained attention due to its association with drug resistance and poor prognosis (1, 2). Notably, lung adenosquamous carcinoma (LUAS), a rare but aggressive biphasic subtype of NSCLC, accounts for 0.7%–11.4% of all NSCLC cases and uniquely exhibits geographically distinct glandular and squamous components (each ≥10% of tumor volume), representing a transitional entity bridging LUAD and LUSC (3). Despite significant advances in understanding cancer biology, the underlying mechanisms driving AST and its impact on immunotherapy remain largely unexplored (1, 2).

Lineage plasticity describes the phenomenon where cancer cells undergo dynamic transformation of their phenotypes during the onset and progression of cancer (2). Through lineage plasticity, tumor cells can shift to different histological subtypes, thereby increasing the heterogeneity of tumors (1) and facilitating immune escape, allowing adaptation to the tumor microenvironment (TME) post-treatment. In clinical practice, following treatment with epidermal growth factor receptor tyrosine kinase inhibitors (EGFR-TKIs) or *KRAS glycine-to-cysteine substitution at codon 12 (G12C)* inhibitors (adagrasib and sotorasib), the transformation of lung AST is frequently observed (4–6). Similarly, LUAD patients also exhibit transformation of lung AST after receiving immunotherapy and chemotherapy (**Table 1**).

**Table 1:** Clinical cases of AST following different treatment regimens.

Treatment pressure	Treatment before the occurrence of AST	Mutation status	References	Note
ALK inhibitor	ALK -TKIs	EML4-ALK variant 2 rearrangement	(7)	
	Crizotinib,Alectinib,Brigatinib,Lorlatinib	EML4-ALK,NFE2L2, KMT2D, MLH1	(8)	
	ALK inhibitor	EML4 - ALK V5 fusion mutation	(9)	
	Lorlatinib	MET amplification	(10)	
	Alectinib	ALK	(11)	High expression of PD-L1
EGFR-TKIs	Erlotinib	EGFR	(12)	without T790M mutation
	Osimertinib	EGFR	(13)	High expression of PD-L1
	Icotinib	EGFR (T790M)	(14)	It was found that most of the transformed patients were female, with an average transformation time of 12.2±5.7 months and an average survival period of 7.1±5.2 months.
	Gefitinib	EGFR (S768I , L858R )	(15)	
	Osimertinib	MET amplification, C797S mutation	(16)	Histological transformation occurred in 5 patients (accounting for 9% of tumor biopsies).
	Erlotinib	EGFR (L858R,T790M)	(17)	
	Osimertinib	EGFR (H835L,L833V,T790M )	(18)	
	Gefitinib	EGFR	(19)	Histological transformation into SCLC and LUSC
	Gefitinib plus Osimertinib	EGFR (T790M)	(20)	
Chemotherapy	cisplatin plus pemetrexed (8 M)		(21)	M(months of treatment)
	Postoperative adjuvant chemotherapy (9 M)		(21)	
	gemcitabine plus cisplatin	EML4-ALK	(22)	
Chemotherapy, EGFR-TKIs	Gefitinib plus chemotherapy plus Osimertinib	EGFR 19 del	(23)	The patient had a low PD-L1 expression level and experienced two histological transformations. First, the LUAD transformed into LUSC, and then it transformed into sarcomatoid carcinoma.
	chemotherapy plus gefitiniband platinum-based chemotherapy	EGFR 19 del	(24)	
	Chemotherapy plus gefitinib plus osimertinib(2Y)	EGFR (T790M),MET amplification	(25)	Y(years of treatment)
	Chemotherapy, EGFR-TKIs	EGFR 19 del	(26)	Histological transformation into a mixed type of SCLC and LUSC
Chemotherapy + Immune Checkpoint Inhibitor(ICIs)	pembrolizumab plus carboplatin plus pemetrexed		(27)	There are no gene mutations in EGFR, ALK, and ROS1, and PD-L1 is not expressed.
Chemo-radiotherapy + ICIs	Pemetrexed plus platinum plus pembrolizumab		(28)	The expression of PD-L1 shows dynamic changes during the treatment process.
	cisplatin/etoposide plus pembrolizumab	BRAF	(29)	

Histologic transformation, such as AST, is a critical resistance mechanism in LUAD. Epidemiologically, AST occurs in up to 9% of EGFR-mutant patients relapsing on osimertinib and contributes to markedly poor prognosis (median survival <6 months post-relapse) (30). Similarly, KRAS G12C-mutant LUAD (13% of all LUAD) treated with adagrasib exhibits AST as a resistance pathway alongside secondary KRAS mutations and RTK-RAS reactivation (5). These transformations are characterized by lineage marker switching (e.g., TTF-1 loss, p63 gain) and activation of pro-squamous transcriptional programs, rendering tumors refractory to lineage-specific therapies. This underscores the need for early detection and adaptive therapeutic strategies, and this transformation is closely linked to immune responses. Interestingly, a study described a 69-year-old never-smoking male NSCLC patient with EGFR/ALK wild-type adenocarcinoma, who developed sequential histological transformations—adenocarcinoma with sarcomatoid change, squamous cell carcinoma with sarcomatoid change, and pure squamous cell carcinoma—during chemotherapy, radiotherapy, and pembrolizumab treatment. PD-L1 expression shifted from positive to negative, highlighting dynamic phenotypic evolution under therapeutic pressure (28). These findings suggest that AST is a key mechanism underlying drug resistance in LUAD treatment.

Recent studies have begun to unravel how AST influences the immune landscape of NSCLC (13), highlighting the role of immune checkpoint inhibitors in modulating the inflammatory response during trans-differentiation. The trans-differentiation from adenocarcinoma to squamous cell carcinoma is not unique to lung cancer but is also prevalent in other organs (31–37). There is a significant correlation between AST triggered by the inactivation of *Liver kinase B1 (LKB1)*, also known as *serine-threonine kinase 11 (STK 11)*, and targeted-therapy resistance (38). It is widely acknowledged that lineage plasticity and immune escape are common mechanisms resulting to acquired drug resistance and subsequent treatment failure.

As two different subtypes of NSCLC, LUAD and LUSC differ in histopathological features, gene expression profiles, and responses to drug treatments (39, 40). Pathological confirmation of LUAS requires strict WHO criteria: (1) morphologically distinct glandular/squamous components via hematoxylin-eosin staining; (2) immunohistochemical validation (e.g., TTF-1/napsin A for adenocarcinoma, p40/p63 for squamous carcinoma); and (3) spatial segregation of components (each ≥10% tumor volume). While small biopsy samples pose diagnostic challenges due to sampling bias, identification of biphenotypic differentiation should prompt molecular or surgical validation (3). Interestingly, approximately 4% to 9% of human NSCLC tumors contain mixed adenomatous and squamous pathologies in a single lesion, clinically termed adenosquamous lung carcinoma (ASLC) (41, 42). While LUAD and LUSC share certain genomic alterations (e.g., TP53 mutations), their overall somatic single-nucleotide variant (SNV) and insertion/deletion (InDel) landscapes exhibit significant divergence, particularly in driver oncogenes and tumor-specific pathways (43). Recent studies have shown that during the AST, an immunosuppressive state is observed in either LUAD or LUSC, whereas their transformed intermediate state exhibits an inflammatory state characterized by increased immune infiltration (3). This implies that the TIME undergoes significant changes during AST, and the understanding of the relevant regulatory mechanisms can help guide the combined application of immunotherapy in clinical practice.

Moreover, common genomic alterations include TP53 and EGFR mutations, as well as gene deletions in the 9q21 chromosomal region, which strongly indicate a common clonal origin for both subtypes (30). This phenomenon implies that there is a potential phenotypic transformation in NSCLC, fully demonstrating that NSCLC has strong cancer plasticity (this plasticity is not limited to lineage plasticity). Additionally, Patients with ASLC usually have poor treatment effects and a poor prognosis (44).

Moving forward, we will delve into the intricacies of the TIME and the molecular mechanisms that drive adenosquamous carcinoma differentiation. We aim to elucidate the role of immune cells in this transformative process and to explore the potential benefits of integrating immunotherapy to improve treatment outcomes for patients with NSCLC.

## 2 Immunotherapy challenges in AST: genetic and immunological factors

*KRAS* stands out as one of the most prevalent mutated genes in lung cancer (45). Among these mutations, *KRAS G12C* inhibitors, such as adagrasib and sotorasib, have presented certain clinical efficacy in the treatment of *KRAS G12C*-mutated lung cancer (46). However, most patients will eventually develop drug resistance during subsequent treatment. For individuals harboring *STK11/LKB1* mutations, tumors exhibiting high-expression of LUSC gene features often respond poorly to adagrasib treatment. *STK11/LKB1* mutations may facilitate the occurrence of AST in tumors by inducing epigenetic plasticity, thereby contributing to resistance to *KRAS* inhibitors (47). Whole-genome sequencing (WGS) results show that in mixed-histology tumors, LUAD and LUSC have a common clonal origin. In addition, changes in TBX3, MET, RBM10, etc. may be related to trans-differentiation, thus affecting the occurrence of AST (30). It’s worth noting that adenocarcinoma subclones might initially emerge and subsequently undergo trans-differentiation into squamous lesions (3). The main driving factor behind the transformation from LUAD to LUSC appears to be transcriptional reprogramming rather than mutational events. During this transformation process, genes related to the PI3K/AKT, MYC, and PRC2 pathways are consistently up-regulated (30). Moreover, the combined activation of PI3K/AKT and MYC can induce squamous features in pre-clinical models of EGFR-mutated LUAD, including mouse models and patient-derived xenograft (PDX) models. Additionally, inhibition of EZH2 or the PI3K/AKT signaling pathway can restore sensitivity to osimertinib in those drug-resistant squamous-like tumors (30).

The Tang team performed WGS and RNA-seq on surgical specimens from 109 LUAS patients (71 males [65.1%], 38 females [34.9%]; median age: 62 years, range: 32–84). The cohort comprised 46.8% non-smokers (lower than Asian LUAD cohorts [58–62.8%]) and predominantly early-stage tumors (Stage I: 50.5%, II: 17.4%, III: 31.2%, IV: 0.9%). Mutational analysis revealed TP53 (59%) and EGFR (43%) as the most frequent alterations, with ALK fusions occurring in 8% of cases. This demographic and clinical profiling underscores the cohort’s relevance to LUAS biology in resectable disease contexts (3). This team identified several potential oncogenic drivers during the development of LUAS, including RAC1, ALK, and AKT1 (3). Through multidimensional scaling analysis of the RNA-seq data of LUAS and LUSC data, LUAS was categorized into three mRNA subtypes: terminal respiratory unit-like (TRU-like), inflammatory, and Basal-like. Among these, the inflammatory subtype features enhanced immune infiltration and serves as an intermediate stage of AST. This implies that cancer cells gradually progress from an adenocarcinoma state to an inflammatory state and ultimately evolve into a squamous state (3). Pathological analysis demonstrates that most tumors exhibit the classic histological pattern of LUAD, while some tumors possess squamous pathological features or mixed pathology. Over time, tumor development transitions from LUAD-dominant LUAS to LUSC-dominant LUAS and finally to typical LUSC, with LUSC tumors being larger in volume (41). The analysis of the mRNA subtype of LUAS identified a dysregulated upstream transcription factor (TFs) network centered around NKX2-1, FOXA2, SOX2, and TP63, which has a regulatory effect on the development of LUAS (3). Furthermore, the research of the Tong team further discovered that the dynamic dysregulation of lineage-specific TFs, including LUAD-related TFs (NKX2–1 and FOXA2) and LUSC-related TFs (ΔNp63 and SOX2), finely tunes the AST process (4). Notably, TTF1 + ΔNp63 + serves as an important marker for AST. The analysis of human ALK-rearranged lung cancer samples also found that some LUADs present squamous features, indicating a tendency towards squamous transformation (41). Clinical analysis revealed that the progression-free survival (PFS) of LUSC patients receiving ALK-TKI treatment is significantly shorter than that of LUAD patients. Moreover, the expression of squamous biomarkers was detected in biopsy samples of recurrent patients, which means that AST is associated with drug resistance (41). Besides, the gene set variation analysis (GSVA) scores of immune cells (such as neutrophils, T-cells, and B-cells) in the inflammatory subtype are significantly higher.

The above mechanisms, including KRAS gene mutations, genetic alterations in mixed-histology tumors, characteristic gene mutations in LUAS, dysregulation of lineage-specific transcription factor expression, and remodeling of the immune microenvironment, collectively induce resistance through a cascade of “genetic variations (e.g., STK11/LKB1, TP53, EGFR mutations)-transcriptional reprogramming (disruption of lineage-specific transcription factor networks and abnormal activation of PI3K/AKT and MYC pathways)-immune microenvironment remodeling (dysfunctional immune infiltration in the inflammatory subtype and the paradox of immune cell accumulation)”. This multi-dimensional resistance is manifested as: 1) intrinsic tumor cell resistance, where cancer cells directly reduce sensitivity to immune checkpoint inhibitors (ICI) through epigenetic plasticity and lineage transdifferentiation; 2) immune microenvironment-mediated resistance, where the “quality” of immune infiltration (e.g., enrichment of immunosuppressive cells or functional exhaustion of effector T cells), rather than just the “quantity” of immune cells, becomes a critical bottleneck for immune therapy response; and 3) unresolved cross-resistance mechanisms between targeted therapies (such as KRAS G12C inhibitors or EGFR tyrosine kinase inhibitors) and ICI, with their interactive roles during AST still requiring in-depth clarification. The convergence of these multiple resistance mechanisms constitutes the complex challenges facing immunotherapy for AST-related lung cancer.

## 3 TFs Networks and signaling pathway alterations in AST

### 3.1 Role of TFs in KRAS-driven ASLC

In *KRAS*-driven LUAD, the TFs networks of LUAD and LUSC can be visualized as a complex seesaw. These networks are intricately balanced, mutually inhibiting each other to maintain a delicate dynamic equilibrium. Disruption of this balance tilts the seesaw towards one side, leading to squamous cell differentiation when the influence of LUSC-related TFs (NKX2–1 and FOXA2) outweighs that of LUAD (p63 and SOX2), ultimately resulting in ASLC (3, 48)

Knockout of NKX2–1 and FOXA2 can drive the occurrence of LUAS, and SOX2 plays a key role in driving squamous trans-differentiation (3). DNp63, the predominant p63 transcript, is overexpressed in tumors compared with normal tissues (49). Through organoid culture of LUAD from *KRAS* ^LSL-G12D/+^/*LKB1* ^flox/flox^ (KL) mice, research has revealed that E74-like factor 5 (ELF5) is crucial for maintaining ADC lineage characteristics and maintaining sensitivity to *KRAS* inhibitors, while ΔNp63 promotes squamous transformation and resistance to *KRAS* inhibitors (4). Elf5 serves as an inhibitor of Vezf1. Upon reducing Vezf1 expression, ΔNp63 expression decreased, and NKX2–1 expression increased. This finding suggests that during the AST process, Elf5 can regulate ΔNp63 expression by modulating Vezf1, potentially inhibiting squamous transformation. Moreover, these alterations in the expression of DNp63 and related genes not only drive AST but also maintain SCC characteristics and lead to resistance to *KRAS* inhibition (47).

In *KRAS*-mutant lung cancer with *LKB1* deletion, Wnt signaling plays a role in maintaining the adenocarcinoma state by activating NKX2-1. However, in the KL mouse model, Wnt signaling is inactivated due to oxidative stress (50). Its downstream effector FOXO3A can be inactivated by reactive oxygen species (ROS). ROS mediates the shutdown of Wnt/β-catenin signaling through FOXO3A, disrupting the balance of the glandular and squamous lineage TF networks, thereby promoting squamous trans-differentiation (51).

In the Rosa26LSL-Sox2-IRES-GFP; *LKB1*fl/fl (SL) mouse model, with overexpression of the TFs Sox2 and loss of the tumor suppressor *LKB1*, the tumors exhibit LUSC characteristics, such as expressing markers like KRT5 and DNp63, and are similar to human LUSC in gene expression and immune infiltration characteristics, including an abundance of tumor-associated neutrophils (TANs), low expression of NKX2-1, and activation of the mTOR pathway (high expression of p4EBP1). In this model, SOX2 inhibits the activity of NKX2-1, which in turn leads to the deletion of NKX2-1 (3, 48). SOX2 can promote the recruitment and infiltration of TANs mediated by CXCL3 and CXCL5 (48), and this phenomenon is independent of the tumor tissue type (48). NKX2–1 functions as a tumor suppressor in LUSC, and its deletion may promote SOX2-driven transformation by inhibiting SOX2 targeted genes, thereby accelerating the development of LUSC (48).

In SNL (deletion of Nkx2–1 in SL mice) mice, the deletion of NKX2–1 significantly accelerates the tumorigenesis driven by the deletion of SOX2 and *LKB1*. The induced tumors are mainly mucinous adenocarcinomas. Over time, these tumors undergo trans-differentiation into LUSC, with an increasing number of ΔNp63+ tumor cells (41). In the KL mouse model, the level of TET-mediated DNA demethylation increases during AST. Knockout of the Tet2 gene indicates its indispensability for squamous carcinoma transformation (36). TET2 promotes lipid transfer of neutrophils through STAT3-mediated CXCL5 expression, thereby promoting the AST process (36). Changes in P63 and its related genes maintain the characteristics of LUSC (46). Targeting STAT3 can significantly reduce the expression of P63 and inhibit the AST process (47). Therefore, targeting STAT3 can enhance the anti-cancer immune response of tumors, rescue the suppressed TIME (52, 53), and simultaneously target the connection between STAT3-CXCL5 to reduce neutrophil infiltration and effectively inhibit squamous transformation (36).

### 3.2 ALK rearrangement and JAK-STAT pathway activation in ASLC

Research findings reveal that *anaplastic lymphoma kinase (ALK)* rearrangement can be detected in 5.1% ~ 7.5% of ASLC. The EML4-ALK G1202R mutation drives Epithelial-Mesenchymal Transition (EMT) via constitutive activation of the STAT3/Slug signaling axis. Mechanistically, G1202R-enhanced ALK kinase activity phosphorylates STAT3, which directly upregulates Slug (SNAI2) expression. Slug orchestrates EMT by repressing epithelial markers (e.g., E-cadherin) and inducing mesenchymal markers (e.g., vimentin, N-cadherin), thereby augmenting tumor cell migration, invasion, and metastatic dissemination (PMID: 35085771). This molecular cascade links ALK mutational activation to both EMT phenotype acquisition and clinically observed aggressiveness in ALK-rearranged tumors (54), indicating that this mutation promotes tumor progression and augments its metastatic potential. Further research has shown that EML4-ALK initially promotes the formation of LUAD and drives squamous transformation in the late stage, altering the morphology and characteristics of the tumor (46, 55). Simultaneously, it has also been found that the JAK-STAT signal activated by EML4-ALK promotes AST, thereby resulting in resistance to ALK inhibitors (46). In addition, research indicates that the EML4-ALK fusion protein may activate the PI3K/AKT signaling pathway to promote the proliferation, survival, and migration of lung cancer cells, thus driving tumor progression. The dysregulation of the PI3K/AKT signaling pathway is relatively common in ASLC, and this dysregulation may be closely associated with tumorigenesis, development, and poor prognosis (56). The well-known inhibitors of the JAK-STAT pathway are ruxolitinib (JAK1/2 inhibitor) and fedratinib (JAK2 inhibitor) (57). Moreover, the combination of JAK1/2 inhibitors with TKI therapy and the combined use of ALK and STAT3 inhibitors can regulate the immune response and restore the sensitivity of tumor cells to treatment (58), thereby overcoming the resistance driven by AST and improving the treatment outcome. In conclusion, the JAK/STAT signaling pathway plays a significant promoting role in the process of LUAD transforming into AST.

### 3.3 The Hippo pathway’s role in mediating AST

The Hippo pathway, along with its downstream effectors, the transcriptional co-activator Yes-associated protein (YAP) and the transcriptional co-activator with PDZ-binding motif (TAZ), can bind to TEADs to regulate gene expression (59). These proteins critical regulatory roles in organ growth, cell plasticity, proliferation, and regeneration (60). In particular, the YAP/TAZ-TEAD transcriptional complex has emerged as a promising target for cancer therapy, with strategies aimed at inhibiting the Hippo pathway by disrupting the YAP/TAZ-TEAD interface (61).

**Figure 1:**
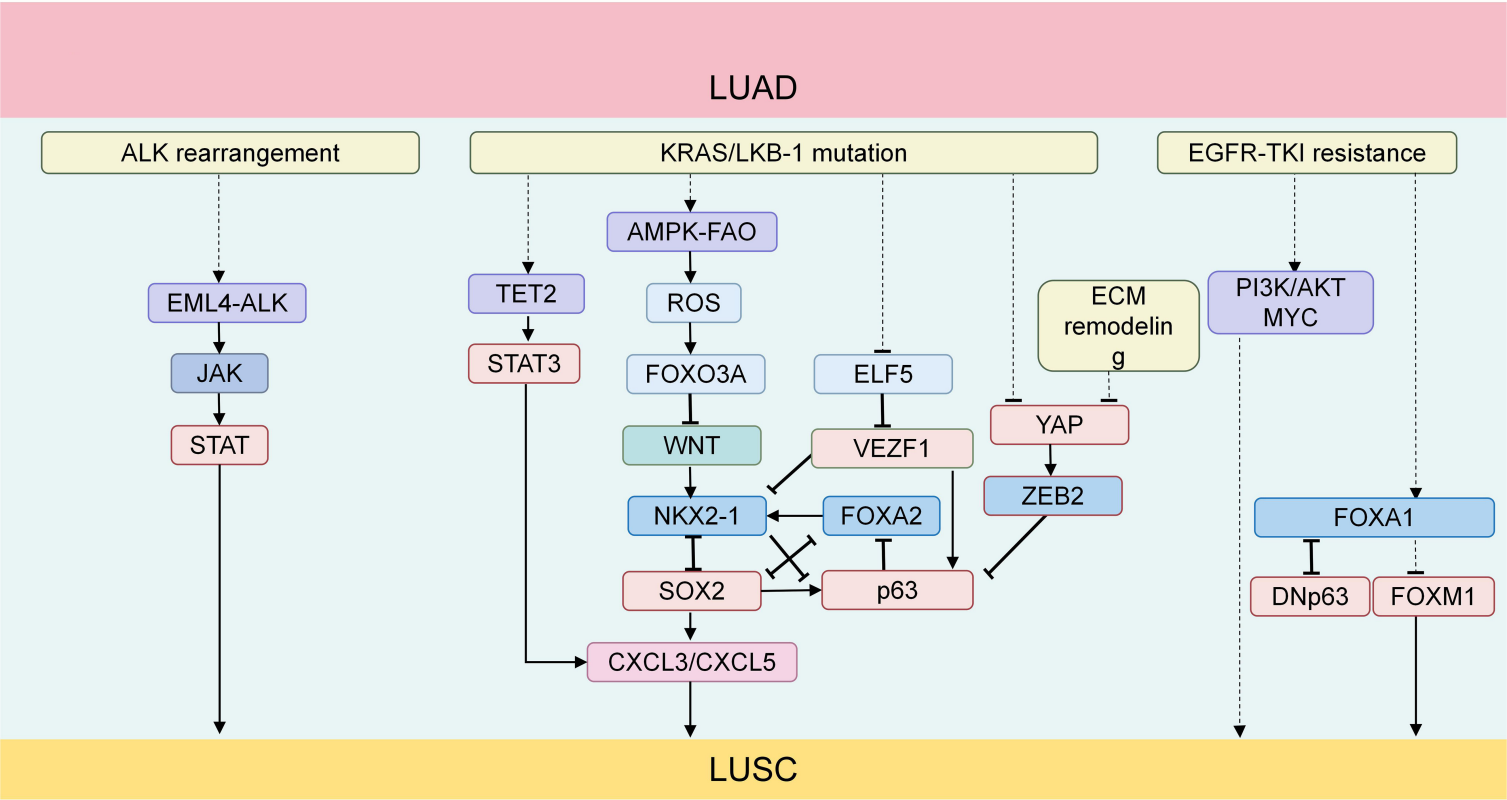
(1) The EML4-ALK fusion protein produced by ALK rearrangement can activate the JAK-STAT signaling pathway. The activated JAK-STAT signaling plays an important promoting role in the process of AST; (2) TET2 promotes neutrophil lipid transfer through STAT3-mediated CXCL5 expression, thus facilitating the process of AST; (3) In KRAS-mutated lung cancer with LKB1 deficiency, the AMPK-FAO pathway is disrupted by the excessively accumulated ROS. Meanwhile, ROS mediates the shutdown of the Wnt/β-catenin signaling pathway through FOXO3A, disrupting the balance of the TF network,thus promoting the development of AST; (4) The upstream TFs of ASLC form a regulatory network centered around NKX2-1, FOXA2, SOX2 and TP63; (5) In KRAS-driven LUAD, SOX2 promotes the recruitment and infiltration of TANs through CXCL3 and CXCL5; (6) YAP inhibits ASLC with LKB1 deficiency by suppressing DNp63 mediated by ZEB2; (7) Activation of the PI3K/AKT pathway can induce squamous characteristics in an EGFR-mutated LUAD model; (8) The deletion of FOXA1and the overexpression of FOXM1synergistically drive AST (63); (9) The knockout of FOXA1 significantly upregulates the transcription of DNp63 in tumor cells. The protein level of FOXA1 is downregulated in tumors with DNp63 overexpression, while it is upregulated in tumors with DNp63 knockout. TFs, transcription factors; LUSC, lung squamous cell carcinoma; LUAD, lung adenocarcinoma; ASLC, adeno-squamous lung carcinoma; TANs, tumor-associated neutrophils; AST, adeno-squamous transformation.

Experimental manipulations of YAP in lung cancer cell lines have revealed that overexpression of YAP results in a marked down-regulation of S100A7 and ΔNp63, alongside a substantial up-regulation of TTF1 and napsin A, effectively inhibiting squamous trans-differentiation. Conversely, knockdown of YAP alone facilitates this trans-differentiation process (59). LSD1 can mediate a large number of genes down-regulated by YAP/TAZ, including tumor suppressors in YAP/TAZ-activated cells, which confirms that YAP/TAZ drives cell proliferation and tumor growth through the polyamine-eIF5A oligomerization-LSD1 axis. In addition, the deletion of *LKB1* leads to YAP activation, causing an up-regulation of ZEB2 expression and inhibiting DNp63 transcription. YAP inhibits *LKB1*-deleted ASLC by inhibiting ZEB2-mediated DNp63 (62). In summary, we have compiled the key factors influencing the balance of TFs in LUAD and LUSC during AST (**Figure 1**).

## 4 LKB1 inactivation: a driver of adenosquamous transformation

*LKB1*, a tumor-suppressor gene, is commonly found to be inactivated in a wide variety of tumor types, with this phenomenon being particularly prominent in LUAD (about 30% of cases) (64). Additionally, *LKB1* serves as a central regulator of chromatin accessibility. *LKB1* mutations account for around 17% in LUAD, and the mutation rate is even higher in ASLC, averaging 39.66%. *LKB1* inactivation can drive AST in lung cancer (49, 65, 66).

Research indicates that in LUAD with *LKB1* deficiency, the reduction in lysyl oxidase (Lox) leads to a decrease in collagen distribution, triggering extracellular matrix remodeling. Simultaneously, the up-regulation of p63 expression prompts LUAD to gradually trans-differentiate into LUSC through pathologically mixed ASLC intermediates (67). Moreover, *KRAS*-mutated lung cancer with *LKB1* deletion exhibits high plasticity (49). Furthermore, *LKB1* deletion results in severe metabolic imbalance and excessive accumulation of ROS.ROS can drive the AST process by disrupting fatty acid oxidation (FAO) mediated by adenosine monophosphate-activated protein kinase (AMPK) (66). ROS can act on various stromal cells, providing metabolic support for tumors, ensuring blood supply, and influencing the tumor’s immune response (68). *LKB1* deficiency also promotes the formation of an immunosuppressive microenvironment and may be associated with primary resistance to anti-PD-1/anti-PD-L1 (64).

Both the oncogene *KRAS* and the tumor-suppressor gene *STK11* play regulatory roles in metabolism, and they are frequently mutated in NSCLC (69). *KRAS*-driven lung cancer often leads to the inactivation of *TP53* and/or *STK11/LKB1* (70). In LUAD patients, *STK11/LKB1* mutations are significantly associated with squamous cell characteristics (44). Recent studies have revealed that in LUAD, tumors with *KRAS/TP53* co-mutations often display significantly elevated PD-L1 expression and an accumulation of cytotoxic T cells, whereas tumors with *KRAS*/*LKB1* (K/L) co-mutations are usually negative for PD-L1 expression and exhibit minimal cytotoxic immune infiltration (71). Lung cancer with K/L deletion is highly invasive (69), lacks PD-L1, and has a poor response to immune checkpoint blockade (ICB) (70).

A comprehensive analysis of factors associated with *LKB1* deletion is pivotal in elucidating the intricate mechanisms underlying the initiation, progression, and heterogeneity of ASLC. This line of inquiry not only deepens our understanding of the molecular foundations of this malignancy but also serves as a cornerstone for the development of targeted therapeutic interventions. Leveraging these insights, we have formulated innovative strategies to address these complexities, as delineated in **Table 2**.

**Table 2:** Immunotherapy strategies for the AST process.

	Discovery/Idea	Therapeutic strategy	Result
LKB1 deficiency	K/L mutant lung cancer, mitochondrial dysfunction in tumor cells, silencing of STING expression, is insensitive to cytoplasmic dsDNA sensing, resulting in the exclusion of T cells from tumor tissue while conferring resistance to PD-L1 blocking therapy	STING agonist/Targeting TREX1/Restore STING expression (72)	Reactivate STING-interferon (IFN) signaling to restore anti-tumor immunity; Re-release STING-IFN signals to recruit T cells and natural killer (NK), cells that are sensitive to NK cell-derived IFN γ;The combination of TREX1 inhibitor and PD-1 can enhance immunogenicity.
	*LKB1* mutant cells have reduced levels of redox-sensitive cofactors and are highly sensitive to HDAC6 inhibition.	HDAC6 inhibitor Combined with glutaminase inhibition (73)	The activity of enzymes originally involved in glycolysis is impaired in *LKB1* mutant tumor cells; The combination of HDAC6 inhibitor and glutaminase inhibition can enhance the killing of *LKB1* mutant tumor cells and anti-tumor efficacy
	In K/L-driven tumors, tumor-intrinsic IFN-γ signaling is impaired.	Dual inhibition of PARP1 and PD-1 (74)	PARP1 inhibition restores disrupted IFN-γ signaling and has a synergistic effect with PD-1 blockade
	The K/L mutation cells were resistant to the MEK inhibitor (trametinib)	HDAC1/3 inhibitor (75, 76)	Using HDAC1/HDAC3 inhibitors can reverse the resistance of *KRAS*/*LKB1* mutant cells to MEK inhibitors (trametinib).
	*LKB1* mutant cancer cells lead to significant inhibition of ICAM1.	CDK4/6 inhibitor (77)	CDK4/6 inhibitors can trigger ICAM1 to coordinate anti-tumor immune responses and make *LKB1* mutant lung cancer sensitive to immunotherapy.
	In K/L-driven tumor cells, LIF signaling can drive tumorigenesis by reprogramming myeloid cells (including granulocytes, monocytes and macrophages) in the TIME.	Target LIF (78)	Targeting LIF signaling can reduce TANs, expand antigen-specific T cells, inhibit tumor progression, and reverse the immunosuppressive tumor microenvironment.
EGFR-TKI	The level of FOXM1 is highly correlated with the prognosis in NSCLC patients with EGFR mutations.	Inhibit β-catenin (79) Such as RCM-1 (80)	Inhibiting β-catenin significantly reversed the gefitinib resistance and invasive phenotype induced by FOXM1.
	Long-term treatment with EGFR-TKIs (such as gefitinib and osimertinib) can induce the transdifferentiation of AST, accompanied by TKI resistance (63).	p-PROTACs (81)	After entering the cells, FOXM1-PROTAC induces the degradation of FOXM1 protein, inhibits the viability, migration and invasion of various cancer cell lines. At the same time, it down-regulates the protein expression levels of the glucose transporter GLUT1 and the immune checkpoint PD-L1, and reduces the glucose metabolism of cancer cells.
		RAPGEF3 inhibitor (63)	For patients with TKI resistance and AST, the combination of RAPGEF3 inhibitor and TKI (such as osimertinib + ESI-09) can overcome the resistance mediated by AST, especially in lung cancers with high RAPGEF3 expression.
		FOXM1 inhibitor (82)	FOXM1 is one of the major TFs that regulate the expression of PD-L1 and modulate the immune response to ICIs. Inhibition of FOXM1 increases the sensitivity of tumor cells to immunotherapy.

STING, Stimulator of interferon genes; TREX1, Three prime exonuclease 1; HDAC6, Histone deacetylase 6; PARP1, Poly ADP-ribose polymerase1; entinostat, HDAC1/HDAC3 inhibitors; CDK4/6, Cyclin-dependent kinase 4 and 6; ICAM1, intercellular adhesion molecule-1; LIF, Leukemia inhibitory factor; p-PROTACs, Peptide proteolysis-targeting chimeras.

## 5 Immune microenvironment dynamics in AST

### 5.1 The role of TANs in AST

The efficacy of immunotherapy is shaped by the dynamic interplay between immune cells and cancer cells within the TIME (83). TIME heterogeneity contributes significantly to tumor metastasis, recurrence, and drug resistance (38, 84). TANs in lung cancer exhibit an activated phenotype, secreting various cytokines and chemokines, and some possess antigen-presenting-cell-like capabilities, stimulating anti-tumor T-cell responses (85). They can also exert anti-tumor effects by directly killing tumor cells and participating in networks that mediates drug resistance. Conversely, TANs can promote tumor growth by driving angiogenesis, remodeling the extracellular matrix, facilitating metastasis, and inducing immunosuppression (86). For example, LUAD cells can activate TANs, upregulating the expression of Notch3 in cancer cells and thereby promoting their own invasion and migration (87). Tumor cells can also induce neutrophils through TGFβ1, activate the Smad2/3 signaling pathway, leading to increased FAM3C production. FAM3C promotes the EMT of tumor cells through the JNK-ZEB1/Snail signaling pathway (88). This interaction enhances the affinity between neutrophils and tumor cells, making TANs an important component of TIME. Locally aggregated TANs may be triggered by external stimuli in the TME and switch between anti-tumor and pro-tumor phenotypes (86). In NSCLC, TANs are more abundant in LUSC than in LUAD and are associated with a poor prognosis (46). In the process of AST, there are often reports of TANs recruitment and infiltration (33, 46, 49, 51, 53). Therefore, the infiltration density of TANs may be a new marker for a poor prognosis in AST. TANs have pro-tumor characteristics and preferentially promote the development of squamous tumors. They may affect the fate of tumor cells by creating a favorable TIME or accelerating adenosquamous transdifferentiation (46).

**Figure 2:**
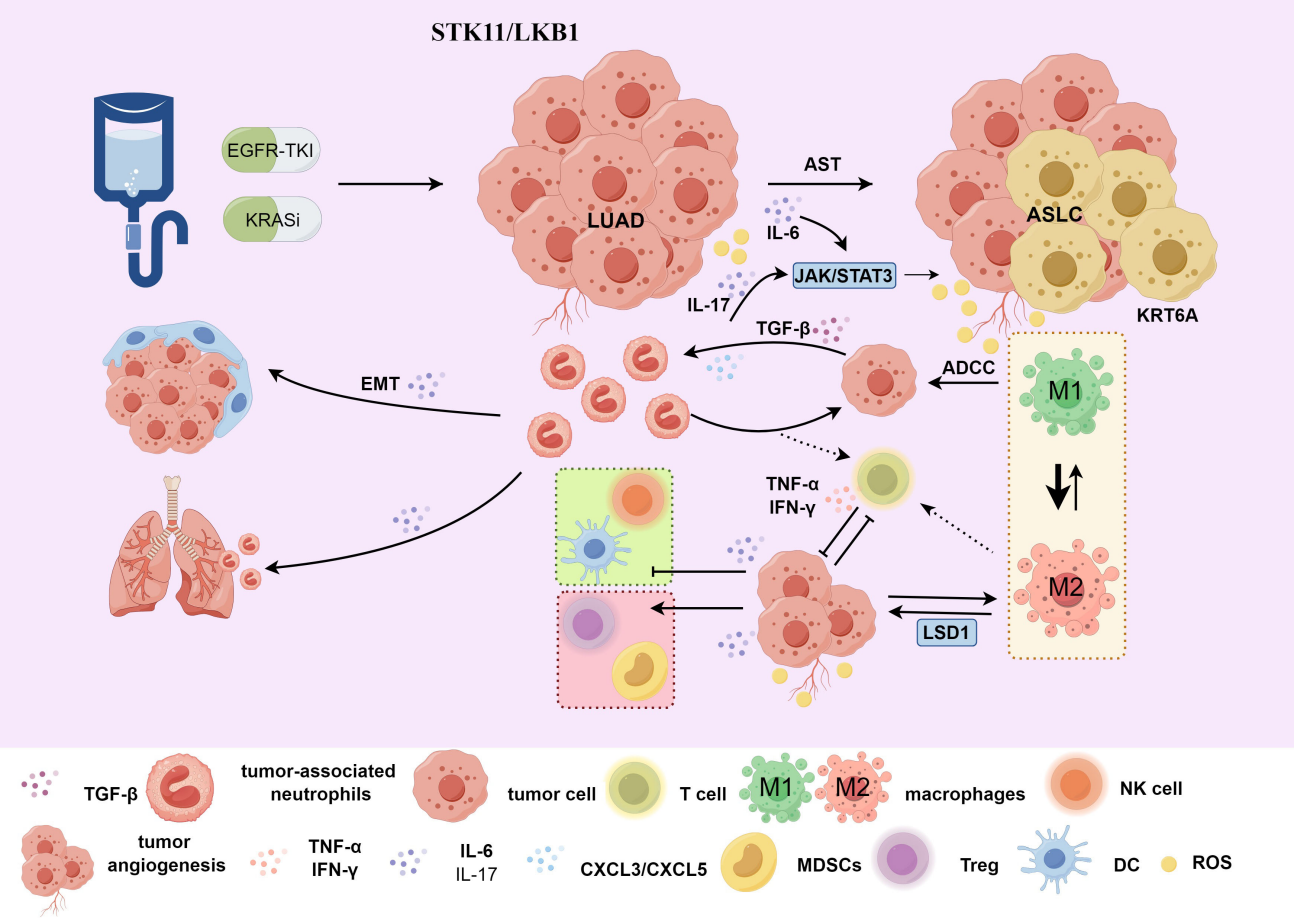
(1) Treatment with EGFR-TKI or *KRAS* inhibitors may lead to AST. For patients with *STK11/LKB1* mutations, the KRT6A gene is highly expressed in the AST transitional state. Under the influence of the local TME, M2-TAMs affects LSD1 and indirectly promotes the tumor cell proliferation and invasion process mediated by KRT6A, affecting treatment prognosis; (2) Tumor cells can induce neutrophils through TGFβ1 to promote EMT. In *KRAS*-driven LUAD, there is recruitment and infiltration of TANs mediated by CXCL3 and CXCL5. TET2 promotes neutrophil lipid transfer via STAT3-mediated CXCL5 expression. TANs inhibit T cell proliferation and reduce the secretion of IFN-γ and TNF-α, affecting the TIME and promoting the AST process; (3) IL-6/IL-17 indirectly influences the TIME of AST through the JAK-STAT signaling pathway, negatively regulating neutrophils, natural killer cells, effector T cells and dendritic cells, and positively regulating regulatory T cells and MDSCs. AST, adenosquamous transformation; M2-TAMs, M2 macrophages; TANs, tumor-associated neutrophils; TIME, tumor immune microenvironment; ADCC, antibody-dependent cell-mediated cytotoxicity; ASLC, adenosquamous lung carcinoma; LUAD, lung adenocarcinoma; EMT, Epithelial-Mesenchymal Transition. Use the website "https://www.figdraw.com/#/" for drawing.

In *KRAS*-driven adenocarcinoma, SOX2 promotes the recruitment and infiltration of TANs through CXCL3 and CXCL5 (46) (**Figure 2**). SOX2 is a TFs related to LUSC and it inhibits the activity of NKX2-1, a TFs related to LUAD. The absence of NKX2–1 significantly accelerates the occurrence of LUSC (41, 46). Consequently, TAN recruitment and infiltration are partially driven by TFs imbalance, facilitating AST. TANs are rich in triglycerides and can transfer lipids to tumor cells, promoting cell proliferation and squamous transformation (33). Inhibiting the STAT3-CXCL5 axis can reduce the infiltration of TANs and thus inhibit squamous cell carcinoma transformation (33). TANs can significantly inhibit T-cell proliferation and reduce the secretion of interferon (IFN-γ) and tumor necrosis factor-α (TNF-α), adversely affecting the TIME (89). Disrupting the balance of TFs in adenocarcinoma and squamous cell carcinoma to promote squamous cell differentiation will have an impact on the infiltration and function of immune cells. The immunosuppressive effect of TANs further worsens this adverse TIME, creating conditions for AST. Intrinsically, the tumor-driving factors SOX2 and NKX2–1 have opposing regulatory effects on TAN accumulation, suggesting that cancer cell intrinsic factors also shape TIME (35), challenging the notion that tissue type alone determines TIME.

In conclusion, AST-driving genes promote the infiltration of TANs, which contributes to oncogenesis through promoting EMT, angiogenesis, inhibiting T-cell activation and killing, and facilitating the formation of an immunosuppressive TIME. Therefore, we hypothesize that the extensive infiltration of TANs may play a crucial role in the transformation of AST from an intermediate state to squamous cell carcinoma. Thus, targeting TANs can effectively inhibit the process of AST.

### 5.2 The promoting role of IL-6/IL-17 pro-inflammatory cytokines on the transformation of TIME

Interleukin-6 (IL-6), produced by various cells within the TME, is a pleiotropic pro-inflammatory cytokine (90). The classic signaling pathway of IL-6 (mIL-6R) is associated with anti-inflammatory functions, while the trans-signaling pathway (sIL-6R) is related to pro-inflammatory responses. The sIL-6R must simultaneously activate the JAK/STAT3 and PI3K/AKT pathways to induce human vascular endothelial cells to release the pro-inflammatory chemokine monocyte chemoattractant protein-1 (MCP-1, also known as CCL2) (91). CCL2 has a dual role: it regulates lymphocyte and NK cell homing, migration, activation, differentiation, and development positively, while also attracting and enhancing other inflammatory factors, promoting monocyte and macrophage infiltration (92–94). CCL2, through its interaction with CCR2, facilitates cancer cell migration and recruits immunosuppressive cells into the TME, fostering cancer progression (95). Therefore, early detection of CCL2 changes can provide insights into the progression of LUAD, and the state of the inflammatory response, particularly in the inflammatory subtype in ASLC. This is helpful for timely adjusting the treatment plan for adenocarcinoma-squamous transformation and further exploring the specific mechanism of CCL2 in AST.

In the TME, the IL-6/JAK/STAT3 signaling can drive the proliferation, survival, invasion, and metastasis of tumor cells and strongly suppress the anti-tumor immune response (96). STAT3 is often over-activated in tumor-infiltrating immune cells, negatively regulating neutrophils, natural killer cells, effector T-cells, and dendritic cells, while positively regulating regulatory T-cells and myeloid-derived suppressor cell (MDSC) populations (96), thereby affecting immune function. The up-regulation of CD47 expression in drug-resistant cells enhances the escape ability of cancer cells. STAT3 is associated with CD47 expression. Inhibiting STAT3 can enhance the phagocytic activity of tumor-associated macrophages (TAMs) and alleviate drug resistance. The combined use of gefitinib, a STAT3 inhibitor, and an anti-CD47 monoclonal antibody can address drug resistance (97). IL-6 activates the JAK/STAT3 signaling in tumor cells and tumor-infiltrating immune cells, participating in the construction of an adverse TIME (91) (**Figure 2**), potentially facilitating AST. Inhibitors targeting IL-6 production, IL-6R, and IL-6 signaling (90) can be used to impede the AST process.

In addition, studies have shown that during acute inflammation, IL-17 can mediate the migration of neutrophils to the lungs, resulting in lung tissue damage (98). The expression of cytokines mediated by IL-17 and the recruitment of neutrophils can trigger tumor proliferation (99) and resistance to immunotherapy (100) in lung cancer. TANs can produce IL-17a and promote the AST process through JAK2/STAT3 signaling (**Figure 2**). Blocking the IL-17a signaling with neutralizing antibodies can inhibit the activity of TANs-stimulated tumor cells (101).

### 5.3 LSD1-mediated TAM polarization in ASLC

Currently, squamous transformation is considered an escape mechanism for adenocarcinoma to evade anti-cancer therapies. However, its key driving factors and molecular alterations have not been fully explored. A clinical trial study showed that squamous transformation occurs in LUAD patients after they develop resistance to the *KRAS* inhibitor adagrasib (48). After analyzing the transcriptomic features and clinical outcomes, it was found that only in LUAD patients, *STK11/LKB1* mutations are significantly associated with squamous cell features and adagrasib resistance, and the keratin type II cytoskeleton 6A (KRT6A) gene is highly expressed during the AST transition state (2, 44). KRT6A expression inversely correlates with treatment duration, predicting poor prognosis in NSCLC patients treated with adagrasib alone, as high KRT6A levels are associated with shorter survival, higher *KEAP1* and *STK11/LKB1* mutation rates, and promote NSCLC cell proliferation and invasion (44, 102, 103).

Mechanistically, KRT6A promotes tumor progression through the pentose phosphate pathway regulated by MYC, and LSD1 can promote KRT6A gene expression (104). The Che team’s research found that KRT6A acts downstream of LSD1 to promote the proliferation and invasion of NSCLC cells (105) In addition, highly expressed KRT6A in LUAD can promote the proliferation and metastasis of lung cancer through EMT and cancer stem cell transformation (106), and can also promote the radioresistance, invasion, and metastasis of lung cancer through the p53 signaling pathway (107).

In summary, KRT6A plays a promoting role in the transformation of LUAD to squamous type, and the promoting effect of LSD1 on KRT6A may directly affect the malignant progression of the tumor. Detecting elevated KRT6A expression early in LUAD can identify AST and adagrasib resistance, aiding in the timely adjustment of treatment strategies to address potential drug resistance.

**Figure 3:**
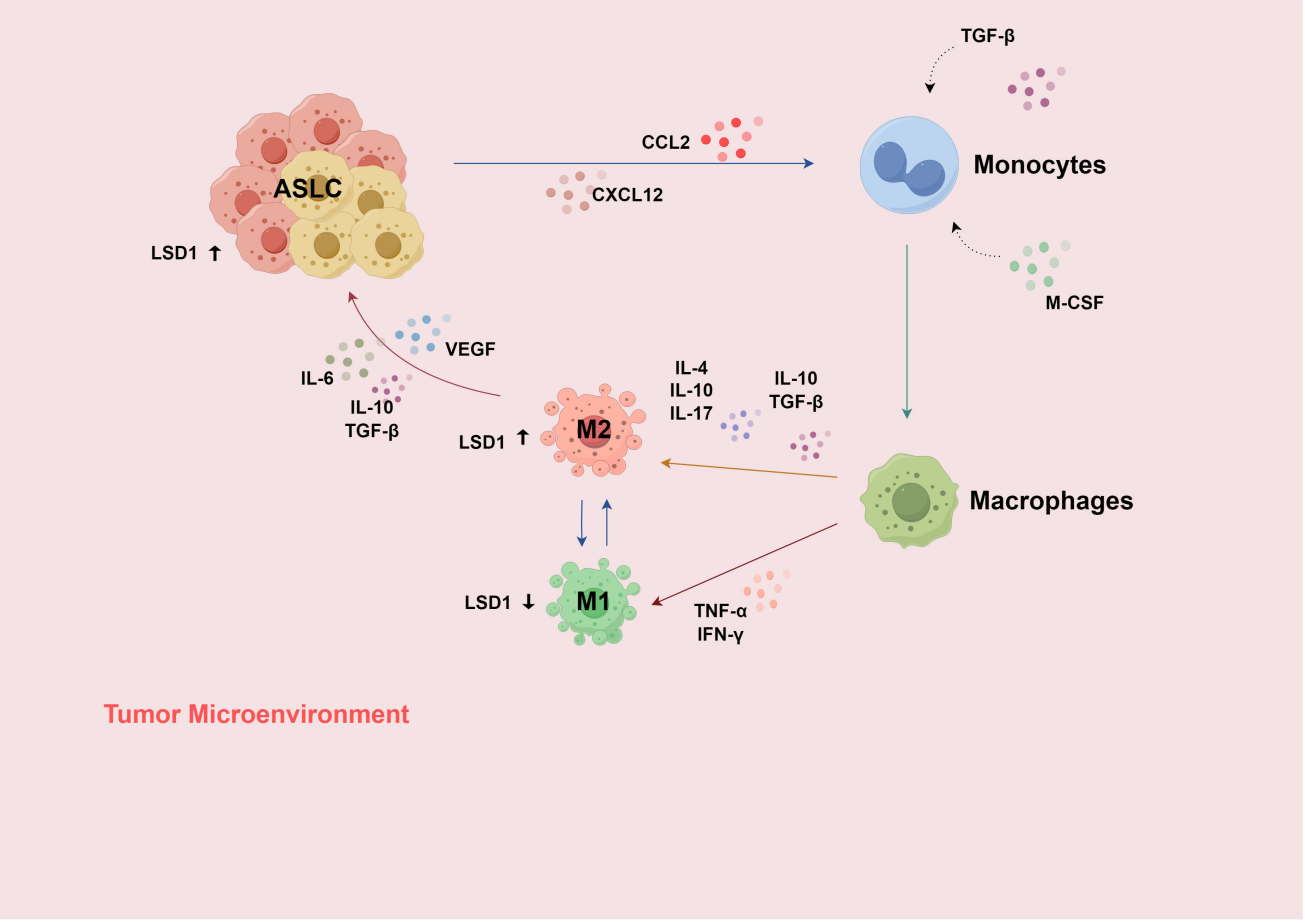
(1) Recruited by the chemokine signals of CCL-2 and CXCL-12, monocytes enter the TME and differentiate into macrophages under the influence of cytokines such as M-CSF and TGF-β; (2) Macrophages are subsequently stimulated by more cytokines in the TME and differentiate into two phenotypes, M1 and M2; (3) When the TME changes or under therapeutic intervention, TAMs can transform into each other; (4) Inflammatory stimulation induced by TNF-α can increase the expression of LSD1 in M2-TAM; LSD1 generates H_2_O_2_ and inhibits CAT, thereby promoting M1 polarization and reducing the expression of LSD1; (5) In the inflammatory subtype of ASLC, immune infiltration increases, and CCL2 promotes the migration and infiltration of macrophages at the site of inflammation. H_2_O_2_, hydrogen peroxide; CAT, catalase; TAMs, tumor-associated macrophages. Use the website "https://www.figdraw.com/#/" for drawing.

Tumors and the TME can affect the pro-metastatic ability of TANs. TAMs, among others, can promote neutrophil-mediated tumor metastasis (46). It is known that inflammatory stimuli promoted by TNF-α can induce an increase in LSD1 expression in M2-TAMs (108). LSD1 promotes pro-inflammatory polarization of human macrophages (M1-TAM polarization) by inhibiting the transcription of catalase (109). During this process, hydrogen peroxide (H_2_O_2_) is produced as a by-product (110). Under normal circumstances, this by-product inhibits catalase, thereby maintaining the M1 polarization state. When an LSD1 inhibitor is used, the catalase level can be maintained (109), preventing LSD1 from promoting M1-TAM polarization. Meanwhile, research has found that M1-TAM polarization, in turn, reduces LSD1 expression (111), forming a feedback-regulation mechanism. Therefore, LSD1 plays a key regulatory role in the TIME of ASLC (**Figure 3**).

Under the influence of the local TME, TAMs exhibit great phenotypic heterogeneity and diverse functional capabilities (112), and can transform into each other when the TME changes or during treatment intervention. The interaction between the TME and cancer cells plays an important role in acquired resistance to EGFR-TKIs (97). Among them, M2-like reprogramming of TAMs and the reduction in macrophage phagocytosis are related to drug resistance (97). TAMs mainly have two functional subtypes. Classically activated M1-TAMs (pro-inflammatory) can directly mediate cytotoxicity and antibody-dependent cell-mediated cytotoxicity (ADCC) to kill tumor cells (113), while M2-TAMs (anti-inflammatory) can promote tumor cell occurrence, metastasis (114), inhibit T-cell-mediated anti-tumor immune responses, and promote tumor angiogenesis, thereby leading to tumor progression (113, 115). (**Figure 2**)

The expression of LSD1 in TAMs is regulated by inflammatory stimuli (57, 116, 117). In turn, LSD1 can regulate the polarization state of TAMs. This forms a complex interaction network impacting the progression and development of ASLC. In summary, LSD1 and TAMs interact through multiple mechanisms in ASLC, having an important impact on tumor progression, the TIME, etc., providing potential targets and research directions for the treatment of ASLC (**Figure 3**).

USP7, highly expressed in M2 macrophages compared to M1, can be silenced with a specific inhibitor, altering M2-TAM phenotype and function. This promotes CD8+ T-cell proliferation and increases PD-L1 expression in tumor cells *in vitro*. The combined use of a USP7 inhibitor and an anti-PD-1 antibody can produce a synergistic anti-tumor effect (118). Manipulating macrophage polarization has emerged as a potential strategy for treating ASLC, either by inhibiting M2-type polarization signals or promoting M1-type polarization to enhance anti-tumor immune responses.

### 5.4 LSD1 inhibition: synergistic potential with immunotherapy in ASLC treatment

Dysregulation of various cell types within the TME can trigger immunosuppressive functions and result in the growth of aggressive tumors (119). LSD1 (lysine-specific histone demethylase 1, also known as KDM1A) is crucial in tumorigenesis and development and has a close connection with immunity (120–122). LSD1 is widely expressed in multiple cancer types. It can block normal cell differentiation, promote the proliferation, migration, and invasion of tumor cells (123), and drive the formation of cancer stem cell phenotypes (124–129). Previous research in ASLC has shown that LSD1, upstream of the highly-expressed KRT6A, promotes KRT6A gene expression, thus facilitating lung cancer cell growth and invasion (105). Depletion of LSD1 enhances the immunogenicity of poorly immunogenic tumors and T-cell infiltration (130), suggesting that inhibiting LSD1 can improve the anti-tumor effect of immunotherapy. Similarly, the Tang group found in the KL model that LSD1 deletion completely blocks the AST process. In this model, only LUAD pathology is present, and the incidence of AST and tumor burden are significantly reduced (3).

H_2_O_2_ is important in the interaction between LSD1 and TAMs in ASLC (57, 116, 117). Superoxide dismutase 2 (SOD2), a mitochondrial superoxide scavenger and H_2_O_2_ regulator, may affect tumor development in the TME by regulating oxidative stress levels. For example, using mitochondrial antioxidants or other methods to regulate SOD2 function can inhibit tumor-associated inflammatory responses and reduce the tumor-promoting effect of inflammation (69). SOD2 promotes the immunosuppressive function of mesenchymal stem cells at the expense of adipocyte differentiation. In an inflammatory microenvironment, it helps regulate the immune response and reduce inflammatory damage (131). The lack of LSD1 activity and the maintenance of PARP1 on the SOD2 promoter significantly increase SOD2 expression. Although LSD1 is considered tumor-promoting, it can also promote M1-TAM polarization. LSD1 and SOD2 may jointly participate in TME regulation. Further study of their interaction mechanism could lead to more effective treatment strategies.

Tolperisone can induce a tumor-suppressive response mediated by the unfolded protein response (UPR) (132). As a potential target for LSD1, it may alter the gene expression profile and biological behavior of tumor cells by regulating LSD1 activity or its demethylation of specific genes. It can also act synergistically with proteasome inhibitors like MG132 to enhance the inhibitory effect on tumor cells. Tolperisone can reprogram M2-TAMs to M1-TAMs, enhancing the immune response in the tumor microenvironment and making the tumor more sensitive to immunotherapy (133). This provides a good basis for further research on combining tolperisone with ASLC treatment.

## 6 Therapeutic strategies targeting key drivers in AST

### 6.1 Targeting TAN in immune microenvironment remodeling

Enhanced immune cell infiltration into the TME may be facilitated by chemokines. Knocking out CXCL3 and CXCL5 can inhibit AST (41). Macropinocytosis (MP) enables tumor cells to extract nutrients from extracellular sources and use them to generate energy (134). Pharmacological inhibition of MP can significantly inhibit the lipid transfer from TANs to cancer cells and block squamous transformation (33). The selective CXCR2 inhibitor SB225002 shows good therapeutic effects by reducing neutrophil infiltration and boosting anti-tumor T-cell activity through CD8+ T cell activation (135). It has been found that granulocyte-macrophage colony-stimulating factor (GM-CSF) derived from tumor cells triggers the expression of the anti-apoptotic Bcl-xL protein and enhances the survival of neutrophils through JAK/STAT signaling. Oral administration of a specific BH3 mimetic (A-1331852) can reduce the survival rate and abundance of TANs and inhibit lung tumor growth without causing neutropenia (136).

### 6.2 Targeting LSD1-mediated epigenetic reprogramming in lineage transdifferentiation

GSK2879552, an irreversible LSD1 inhibitor, has been reported to effectively suppress small-cell lung cancer (SCLC) (137). Treatment of the KL model with GSK2879552 significantly inhibits KL tumor progression and neutrophil infiltration. Most mice show ADC pathology, and the incidence of AST and tumor burden are also greatly decreased (127), highlighting LSD1 as a potential therapeutic target for *STK11*-mutated ASLC.

Interestingly, inhibiting LSD1 upregulates PD-L1 expression on tumor cells, affecting the response to immune checkpoint inhibitors (117). In related studies, PD-L1-mediated *in-vivo* T-cell immunity in exosomes can regulate tumor cell proliferation. LSD1 deletion reduces PD-L1 expression in exosomes and restores T-cell responses (127). Similarly, in the NSCLC xenograft model, the combination of an LSD1 inhibitor and a ferroptosis inducer has a stronger anti-tumor effect than either drug alone (116). The Mamun team’s research also confirms the potential of combining LSD1 inhibitors with ICB therapy in future cancer research (119), providing new directions for ASLC treatment.

## 7 Conclusion

In-depth exploration of the changes in key signaling molecules and multiple signaling pathways during the transformation process of ASLC holds irreplaceably significant implications for further unveiling the mystery of the lung cancer development mechanism and for mining potential therapeutic targets. This review has systematically presented the molecular and immunological landscape of AST, highlighting the intricate interplay between lineage plasticity, immune escape, and treatment efficacy.

Our analysis revealed that *LKB1* inactivation in tumors, particularly in LUAD and ASLC, significantly drives AST through mechanisms involving extracellular matrix remodeling, metabolic imbalance, and the formation of an immunosuppressive microenvironment. These findings underscore the pivotal role of *LKB1* in maintaining lung cancer lineage and suggest that targeting *LKB1*-related pathways could be a promising therapeutic strategy for ASLC. The imbalance between TFs like SOX2 and NKX2–1 was identified as a central driver of AST. The dynamic dysregulation of lineage-specific TFs finely tunes the AST process, indicating that targeting these networks could potentially halt or reverse squamous transformation. The inflammatory subtype of ASLC, serving as an intermediate stage of AST, exhibits increased immune cell infiltration. This suggests that the TIME undergoes significant changes during AST, potentially offering a window for immunotherapy intervention. The role of pro-inflammatory cytokines like IL-6 and IL-17 in promoting the transformation of the TME was also highlighted, suggesting a dual role in both inflammation and immune modulation.

The phenomenon of AST profoundly impacts cancer treatment efficacy, revealing the complexity and challenges in managing lung cancer. Our findings emphasize the urgent need to explore immunotherapy strategies that account for lineage plasticity and immune escape. For instance, the observed association between *STK11/LKB1* mutations and resistance to *KRAS* inhibitors in LUAD patients with squamous features provides a critical clue for understanding drug resistance mechanisms. While previous studies have noted the association between lineage plasticity and drug resistance, our review provides a comprehensive overview of how AST influences the immune landscape of NSCLC. We link specific genetic alterations, like ALK rearrangements and the JAK-STAT pathway, with AST and resistance to targeted therapies, offering a more nuanced understanding than previously reported.

One limitation of this review is the reliance on preclinical models, which may not fully capture the heterogeneity of human tumors. The complexity of the TME and the dynamic nature of immune cell interactions within it pose challenges in translating findings into effective clinical interventions. Furthermore, while we propose several potential therapeutic strategies, their efficacy and safety in clinical settings require further validation. Future research should focus on comprehensive molecular profiling to identify predictive biomarkers for AST transitions, further investigation into the molecular mechanisms driving lineage plasticity, particularly the roles of TFs and their regulatory networks, development of combination therapies that target both the tumor cells and the TIME to enhance treatment efficacy and overcome resistance, exploration of targeted therapies based on key signaling pathways identified in this review, including LSD1 inhibitors and other epigenetic modifiers, and employing single-cell technologies to dissect tumor heterogeneity and immune cell dynamics within ASLC.

The evolving understanding of lung cancer heterogeneity demands focused efforts on LUAS and AST. Future research should prioritize: (1) single-cell multi-omics (scRNA/ATAC-seq) to resolve LUAS plasticity and immune-microenvironment crosstalk; (2) AST-focused trials targeting high-risk subgroups (e.g., KRAS/EGFR-mutant LUAD with LKB1 loss) with ctDNA-guided monitoring; (3) multi-omics harmonization bridging genomics (TP53/EGFR), proteomics, and preclinical models.

In summary, the interplay between signaling pathways, immune cell dynamics, and the TME in ASLC, particularly during the AST process, requires a nuanced understanding. This review underscores the need for personalized treatment strategies that address not only the tumor cells but also the supportive microenvironment, thereby improving treatment outcomes and overcoming resistance. By integrating these insights, we can move towards more effective and tailored therapeutic interventions for ASLC.
